# Different role of the gut microbiota in postmenopausal and senile osteoporosis

**DOI:** 10.1038/s41413-025-00432-1

**Published:** 2025-06-13

**Authors:** Xuan-Qi Zheng, Zhi-Yuan Guan, Yun-Di Zhang, Chun-Li Song

**Affiliations:** Department of Orthopaedics, Peking University Third Hospital, Beijing, China

**Keywords:** Metabolomics, Bone

**Fig. 1:**
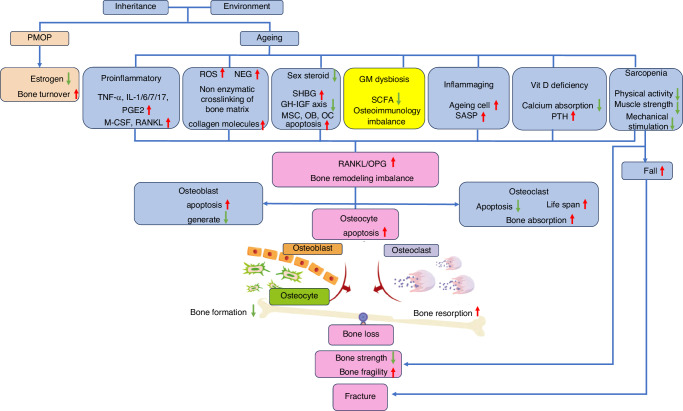
Pathogenesis of primary osteoporosis.^11^ PMOP Postmenopausal osteoporosis; ROS Reactive oxygen species; NEG nonenzymatic glycosylation; SHBG Sex hormone-binding globulin; GM Gut microbiota; SCFA short chain fatty acids; Rankl, Receptor Activator of Nuclear Factor-κ B Ligand; OPG Osteoprotegerin; OB osteoblast; OC osteoclast; M-CSF macrophage-colony stimulating factor; TNF-α tumor necrosis factor α; PGE2: Prostaglandin E2; IL-1/6/7/17, Interleukin (IL)-1/6/7/17; ↑: upgrade; ↓, downgrade

Gut microbiota (GM) exerts an indispensable effect in human health, especially in metabolism regulation.^1^ Recent studies have identified a potential association with osteoporosis.^2–5^ At the same time, GM intervention, such as antibiotic treatment,^6^ fecal microbiota transplantation (FMT),^7,8^ supplement of probiotics^9,10^ and other means, will also subsequently affect bone metabolism. Further, GM dysbiosis was also mentioned as one of pathophysiological mechanism of osteoporosis, even written in the clinical diagnosis and treatment guidelines^11^[Fig. 1, based on Guidelines for the diagnosis and treatment of primary osteoporosis in China (2022)].

Now, *You* et al. have conducted a preclinical study based on germ-free (GF) mice.^12^ They found that bone loss with aging is independent of gut microbiome in CB6F1 mice. Specifically, after 23 months treatment (remain GF or colonization with microbiome from 3-month-old male donors), there were no significant differences in trabecular bone volume fraction, cortical thickness, or cortical area between the GF and colonized mice in either sex.^12^ Another important finding was that the effect of microbial colonization on bone phenotype was independent of the age of the fecal donor (Table 1, based on You’s study). These results have important implications for understanding the gut-bone axis, especially in the aged-related bone loss field.

**Table 1:** The effect of microbial colonization on bone phenotype

	Female				Male			
	BV/TV (in femur)	Cortical thickness	Serum bone formation	Serum bone resorption	BV/TV (in femur)	Cortical thickness	Serum bone formation	Serum bone resorption
With/without GM (GF *vs* Col)	-	-	↑	-	-	-	↑, *P* = 0.07	-
Donor age (3 months *vs* 24 months)	-	-	↑, *P* = 0.12	↓	N.A	N.A	N.A	N.A
Short-term Col (GF *vs* 1 month Col)	↓	-	↑	↑	N.A	N.A	N.A	N.A
Long-term Col (GF *vs* 8 months Col)	-	-	-.	-	-	-	N.A	N.A

*BV/TV* Bone volume/tissue volume; *Col* colonization, *N.A* not available; -, no significance; ↑: upgrade; ↓, downgrade

This pivotal study prompts us to consider whether GM exhibited divergent influences in senile and postmenopausal osteoporosis. Our previous studies demonstrated that estrogen deficiency-induced bone loss is GM-dependent.^3^ Specifically, we show that ovariectomized mice exhibited GM dysbiosis and high levels of lipopolysaccharide. After GM depletion through antibiotic treatment, the bone mass in ovariectomized mice improved. One of the possible explanations is that the metabolism of estrogen is related to the GM.^13,14^ *You*’s study presents new evidence about senile osteoporosis and GM, thus inspires further research.^2^ In fact, clinical data based on cohort studies provide direct evidence of patients with senile osteoporosis^15,16^ and postmenopausal osteoporosis,^17,18^ and found that the two showed heterogeneity in the composition of GM. Consequently, the function/role of the GM in the different osteoporosis progress may also be different. Therefore, more evidences are required for aging and GM went beyond sequencing.

Besides, in *You’s* study, the distal femur was considered as a region of interest, and its bone microstructure was analyzed and demonstrated.

**Fig. 2:**
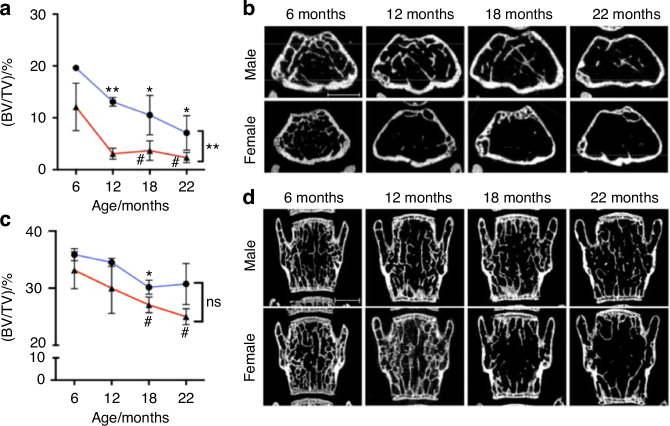
Micro‑computed tomography assessment of bone structure in aging mice.^19^ **a** Quantification of morphometric parameters of the femoral trabecular bone of males (blue lines) and females (red lines); trabecular bone volume fraction (BV/TV). **b** 2D cross-sectional images of trabecular bone in the femur of male and female mice at different ages. **c** Quantification of morphometric parameters of the L3 vertebrae trabecular bone of males (blue lines) and females (red lines); trabecular bone volume fraction (BV/TV). (D).2D cross-sectional images of trabecular bone in the femur of male and female mice at different ages

As a matter of fact, ageing mice exhibit different degrees of site-specific bone loss in the lumber vertebra and distal femur^19^ (Fig. 2, based on Shim’s study). However, the impact of FMT on the vertebral body of GF mice was not previously investigated, either in *You’s* study or by others. This is the direction of future research, and this may have implications for our comprehension of the mechanisms by which GM regulate systemic bone metabolism.

Additionally, another interesting thing is that hip fracture and vertebral fractures are the two typical types of osteoporotic fractures. However, the incidence of hip fractures among people aged 50 years and older in urban China is stable,^20^ while the incidence of vertebral fractures continues to increase.^21^ Thus, the vertebral fracture with more bone trabeculae may truly reflect the progression of osteoporosis.

**Fig. 3:**
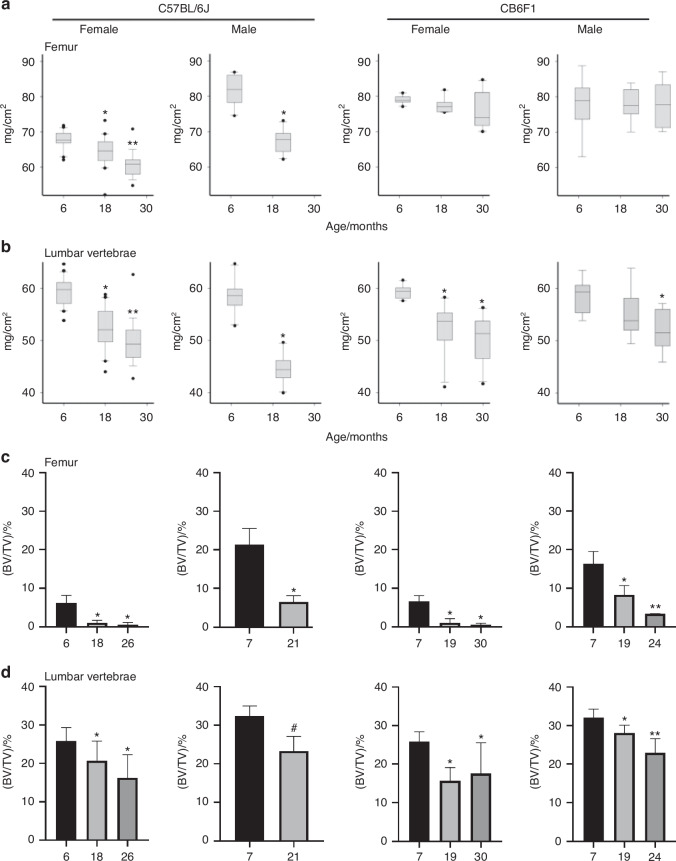
Age-related bone loss is greater in C57BL/6 J than in CB6F1 mice. **a**, **b** Bone mineral density was determined in the femur, lumbar vertebrae. Trabecular architecture was measured by micro-CT of the femur (**c**) and lumbar vertebra L4 (**d**). Trabecular bone volume (BV/TV) were shown in this figure

It is worth noting that the experimental subjects used in *You’s* study was CB6F1 germ-free mice generated from female BALB/c and male C57BL/6 mice. The CB6F1 mouse strain is widely used in cancer or transplant related research for its characteristics in the immune response. However, age-dependent bone loss differs in C57BL/6 J and CB6F1 mice. In particular, the vertebral BMD decreased with age in both males and females of both strains, but BMD in the femur decreased only in C57 BL/6J^22^ (Fig. 3, based on Marilina’s study). The results suggest that genotypes and animal background significantly influence bone development, bone growth, and age-related bone loss. The results also confirm that age-related bone loss is site specific.

In conclusion, the function of GM is subject to variation depending on the specific osteoporosis model under consideration, and the effects of GM may also be site-specific. Despite the extensive research conducted on GM in the context of other diseases, both preclinical and clinical, there remains a paucity of knowledge regarding the gut-bone axis.
